# Neuropeptide S Receptor Stimulation Excites Principal Neurons in Murine Basolateral Amygdala through a Calcium-Dependent Decrease in Membrane Potassium Conductance

**DOI:** 10.3390/ph14060519

**Published:** 2021-05-27

**Authors:** Sion Park, Pia Flüthmann, Carla Wolany, Lena Goedecke, Hannah Maleen Spenner, Thomas Budde, Hans-Christian Pape, Kay Jüngling

**Affiliations:** Institute of Physiology I, Westfälische Wilhelms-Universität Münster, Robert-Koch Strasse 27a, 48149 Münster, Germany; sion.park@hotmail.com (S.P.); piafluthmannde@gmail.com (P.F.); carla.v.wolany@icloud.com (C.W.); Lena.Goedecke@web.de (L.G.); hannahspenner@uni-muenster.de (H.M.S.); Thomas.Budde@ukmuenster.de (T.B.); papechris@ukmuenster.de (H.-C.P.)

**Keywords:** NPSR1, amygdala, potassium conductance, calcium, patch-clamp, mice

## Abstract

Background: The neuropeptide S system, consisting of the 20 amino acid neuropeptide NPS and its G-protein-coupled receptor (GPCR) neuropeptide S receptor 1 (NPSR1), has been studied intensively in rodents. Although there is a lot of data retrieved from behavioral studies using pharmacology or genetic interventions, little is known about intracellular signaling cascades in neurons endogenously expressing the NPSR1. Methods: To elucidate possible G-protein-dependent signaling and effector systems, we performed whole-cell patch-clamp recordings on principal neurons of the anterior basolateral amygdala of mice. We used pharmacological interventions to characterize the NPSR1-mediated current induced by NPS application. Results: Application of NPS reliably evokes inward-directed currents in amygdalar neurons recorded in brain slice preparations of male and female mice. The NPSR1-mediated current had a reversal potential near the potassium reversal potential (E_K_) and was accompanied by an increase in membrane input resistance. GDP-β-S and BAPTA, but neither adenylyl cyclase inhibition nor 8-Br-cAMP, abolished the current. Intracellular tetraethylammonium or 4-aminopyridine reduced the NPS-evoked current. Conclusion: NPSR1 activation in amygdalar neurons inhibits voltage-gated potassium (K^+^) channels, most likely members of the delayed rectifier family. Intracellularly, G_αq_ signaling and calcium ions seem to be mandatory for the observed current and increased neuronal excitability.

## 1. Introduction

The neuropeptide S system, consisting of the 20 amino acid neuropeptide NPS and its G-protein-coupled receptor (GPCR) neuropeptide S receptor 1 (NPSR1) has been identified in the central nervous system of rodents and humans [1,2]. NPS-expressing neurons seem to be largely restricted to distinct brain stem nuclei, located in the pericoerulear region and between the lateral parabrachial and Koelliker–Fuse nucleus in mice [3,4]. In contrast, NPSR1-expressing neurons are found in a variety of regions within the central nervous system of rodents, e.g., olfactory areas, amygdala, frontal and retrosplenial cortex, and midline thalamic regions [3]. Pharmacological studies and use of NPS- or NPSR1-deficient mice point to an important role of the NPS system, e.g., in memory formation [5,6], fear and anxiety [7,8,9,10], social behavior [11], addiction [12,13], and arousal/attention [1,5].

GPCR-mediated signaling can be G protein dependent or, according to more recent concepts, independent [14]. G proteins can be subdivided into four major families: G_q/11_, G_S_, G_i/o_, or G_12/13_, all triggering different intracellular signaling cascades when activated [15]. Upon activation, the heterotrimeric G protein dissociates into an α and a β/γ subunit, each able to modulate intracellular or membrane-located effectors. G protein signaling is initiated by the exchange of GDP by GTP, and is terminated when GTP is hydrolyzed to GDP by the α subunit [15]. In addition to G proteins, activated GPCRs can couple to arrestins or G-protein-coupled receptor kinases (GRK), which, in turn, can contribute to intracellular signaling cascades or lead to GPCR desensitization and internalization.

Early studies in HEK and CHO cell lines expressing human or murine NPSR1 showed that NPSR1 activation by its ligand triggers intracellular signaling cascades involving an increase in intracellular calcium levels, formation of cAMP, and MAPK phosphorylation [16,17,18]. These findings strongly suggest that NPSR1 activation is followed by signaling cascades involving G_q_ and G_s_. In addition, calcium transients following NPSR1 activation have been observed in neurons, and pharmacological interventions strongly suggest pathways dependent on phospholipase C (PLC), inositol-3-phosphate receptors (IP_3_R), and ryanodine receptors (RyR) to be involved in NPSR1 signaling [19,20,21].

Electrophysiological recordings in neuronal slice preparations revealed inward-directed currents, increased neuronal excitability and enhanced synaptic activity following NPSR1 activation [8,21,22,23,24]. With few exceptions (e.g., [21]), a link between the observed modulation of ionic currents and the underlying signaling cascades is still largely lacking.

Here we used a straight-forward electrophysiological approach to identify the ionic nature of the NPSR1-mediated current in principal neurons of the anterior basolateral amygdala (aBA PNs), which have been implicated in processes of fear and extinction [24]. We identified putative intracellular signaling pathways following NPSR1 activation, consequently inducing a reduction of K^+^ conductances, and thereby increasing neuronal discharges.

## 2. Results

### 2.1. NPS-Induced Inward Currents in NPSR1-Expressing aBA Principal Neurons

**Figure 1:**
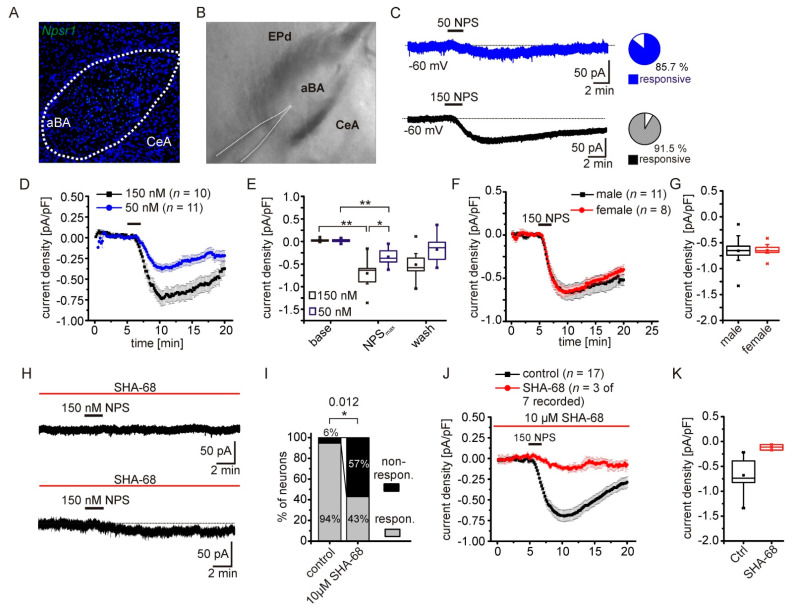
NPS-induced inward current in NPSR1-expressing aBA principal neurons. (**A**) *Npsr1* mRNA (green) detection in neurons of the aBA by fluorescence in situ hybridization. DAPI (blue) was used to mark cellular nuclei. The medially located central nuclei of the amygdala (CeA) are devoid of *Npsr1* mRNA. (**B**) Example of a coronal slice preparation used for electrophysiological recordings of aBA PNs. The white lines delineate the recording pipette. (**C**) Examples of membrane currents recorded from aBA PNs at a holding potential of -60 mV. NPSR1-dependent inward-directed currents were elicited by application of 50 nM (blue) or 150 nM (black) NPS. Pie charts show the overall fraction of NPS-responsive neurons during application of 50 or 150 nM NPS. (**D**) Time course of mean current densities elicited by 50 (blue) or 150 nM (black) NPS. NPS was applied for 2.5 min. (**E**) Quantification of recorded current densities following application of either 50 or 150 nM NPS. The maximal current densities are concentration dependent. (**F**) Time course of the NPS-induced current recorded in aBA PNs in slices from male and female mice. No sex-dependent differences were detected in the time course or in the maximal current densities (**G**). (**H**) Examples of 150 nM NPS application in the presence of the NPSR1 antagonist SHA-68 (10 µM). Examples depict nonresponsive (upper trace) and responsive neurons (lower trace). (**I**) Fraction of responsive aBA PNs in the presence of SHA-68 and during control conditions. (**J**) Mean time course of responsive neurons in the presence of SHA-68 (*n* = 3) and during control recordings. (**K**) In the presence of SHA-68, the maximal current density was significantly reduced. * *p* < 0.05; ** *p* < 0.01.

As described previously [3,8,24], we detected NPSR1-coding mRNA predominantly in the anterior part of the basolateral amygdala (aBA) by fluorescence in situ hybridization in mouse brain slices (Figure 1A). A second population of NPSR1-expressing principal neurons (PN) is located in the lateral nucleus of the amygdala (LA; [24]), but the posterior regions of the basolateral nucleus and the central nucleus of the amygdala (CeA) are largely devoid of NPSR1 mRNA. We performed whole-cell voltage-clamp recordings from PNs in coronal slices containing the aBA (Figure 1B) to analyze intracellular signaling cascades following NPSR1 activation by NPS. Application of 50 or 150 nM NPS elicited a transient inward current in aBA PNs (Figure 1C). The overall fraction of NPS-responsive neurons was 85.7 and 91.5 % following 50 or 150 nM NPS, respectively. The current density evoked by 150 nM was significantly larger compared to current densities evoked by 50 nM (150 nM NPS: −0.70 ± 0.09 pA/pF; 50 nM NPS: −0.42 ± 0.04 pA/pF; RM-ANOVA interaction of time and concentration: F(2,38) = 6.8; *p* = 0.003; Bonferroni post hoc: 150 baseline vs. 150 max: *p* = 1.3 × 10^−11^; 50 baseline vs. 50 max: *p* = 1.3 × 10^−5^; 150 max vs. 50 max: *p* = 0.032; Figure 1D,E). Since male and female mice were used, we analyzed the current density in both sexes in a separate set of experiments (Figure 1F,G). Neither the time course nor the maximal current density differed between male and female mice (males: −0.657 ± 0.09 pA/pF; *n* = 11; females: −0.656 ± 0.05 pA/pF; *n* = 8; *t*-test: *t* = −0.09; df = 17; *p* = 0.926). In the following, data from both sexes were pooled. During voltage-clamp recordings, the input resistance of the cell was monitored by applying brief voltage steps (−5 mV; 50 ms duration) in each recorded sweep (10 s duration). Under baseline conditions, the input resistance was 167 ± 6 MΩ, and increased significantly in the presence of 150 nM NPS to 208 ± 8 MΩ (paired *t*-test: *t* = −7.82; df = 36; *p* = 2.8 × 10^−9^; *n* = 37).

To test the specificity of our approach, we applied NPS in the presence of the NPSR1-specific antagonist SHA-68 ([25]; Figure 1H). The fraction of non-responsive neurons significantly increased to 57 % in the presence of SHA-68, compared to 6 % under control conditions (Fisher’s exact test: *p* = 0.012; Figure 1I). Only three of the recorded aBA PNs showed a detectable inward current upon NPS application, which was significantly decreased compared to controls (control: −0.679 ± 0.07 pA/pF; *n* = 17; SHA-68: −0.116 ± 0.032; *n* = 3; *t*-test: *t* = −3.28; df = 18; *p* = 0.004; Figure 1J,K). These data substantiate the evidence that NPSR1 activation by NPS induces a transient inward-directed current in aBA PNs of male and female mice. In this way, NPS could excite the neurons and activate amygdala networks. 

### 2.2. The NPSR1-Dependent Current Results from Reduced Potassium Conductances

**Figure 2:**
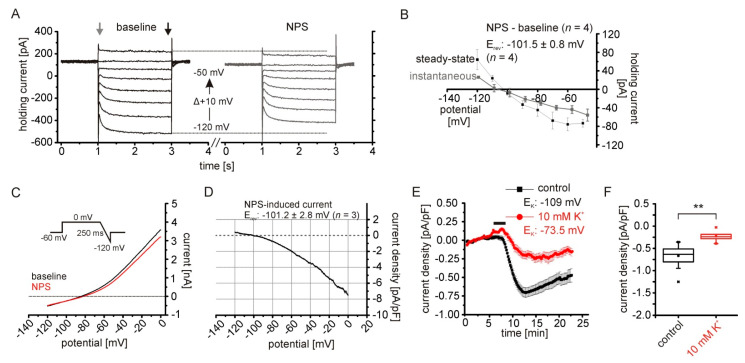
The NPSR1-dependent current results from reduced potassium conductances. (**A**) Voltage steps (−120 mV to −50 mV; Δ + 10 mV; 2 s duration) from a holding potential of −60 mV in the voltage-clamp mode. Recordings were done under baseline conditions and in the presence of 150 nM NPS. For both conditions, baseline and in the presence of NPS, the steady-state current (black arrow) and the instantaneous current (grey arrow) were analyzed. (**B**) Plot of the I–V curves calculated from step protocols. The NPS-induced current was calculated as: current_NPS_-current_baseline_. (**C**) Example of hyperpolarizing ramp recordings (0 mV to −120 mV; 250 ms) from aBA PNs during baseline conditions (black) and in the presence of 150 nM NPS (red). (**D**) Mean NPS-induced current calculated from three recordings. The resultant current was calculated: current_NPS_-current_baseline_. (**E**) Time course of the NPS-induced current during control conditions (black), and with elevated potassium concentration (10 mM; red) in the ACSF. (**F**) In the presence of 10 mM K^+^, the NPS-induced current is significantly reduced compared to control conditions. ** *p* < 0.01.

To characterize the nature of the NPSR1-mediated current, hyper- and depolarizing voltage steps with increasing amplitudes were applied from a holding potential of −60 mV (Figure 2A). The NPS-induced current was calculated at each voltage step by subtraction of the current obtained during baseline. Amplitudes of the instantaneous current at the beginning and the steady state current at termination of the voltage step were analyzed, and the calculated NPS-induced current was plotted against the respective step potential (Figure 2B). The reversal potential of the NPSR1-mediated current was −101.5 ± 0.8 mV (*n* = 4) for steady state currents and, thus, was close to the calculated K^+^ reversal potential (E_K_) of −109 mV. To confirm these findings, hyperpolarizing voltage-clamp ramps were performed from 0 mV to −120 mV in 250 ms (Figure 2C). The NPS-induced current calculated by subtraction of ramp_NPS_–ramp_baseline_ had a reversal potential of −101.2 ± 2.8 mV (*n* = 3), again close to E_K_ (Figure 2D). Therefore, we reasoned that the NPSR1-mediated inward current is due to a reduction of a K^+^ conductance. Therefore, reducing the electrochemical gradient for K^+^ should affect the amplitude of the NPSR1-mediated current recorded at −60 mV. We shifted E_K_ from −109 mV to −73.5 mV by increasing extracellular K^+^ from 2.5 to 10 mM, thereby decreasing the electrochemical driving force from approximately −49 mV to −13.5 mV. The current density of the NPS-induced current was reduced to −0.23 ± 0.05 pA/pF (*n* = 6) in 10 mM K^+^, and was significantly smaller than under control conditions (control: −0.668 ± 0.06 pA/pF; *n* = 15; *t*-test: *t* = −4.31; df = 19; *p* = 3.8 × 10^−4^; Figure 2E,F).

### 2.3. The NPS-Induced Current Is Dependent on NPSR1-Gα-Signaling

**Figure 3:**
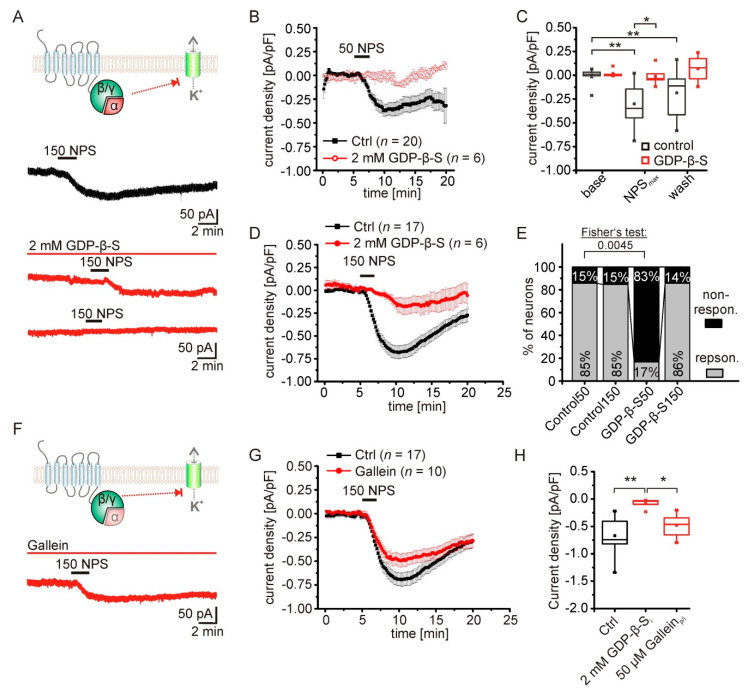
The NPS-induced current is dependent on NPSR1-Gα-signaling. (**A**) To differentiate between G-protein-dependent and direct GPCR-effector interactions leading to changes of potassium conductances, GDP-β-S was used to inhibit intracellular G protein signaling. Example traces show the NPS-induced current during control conditions and with 2 mM GDP-β-S in the intracellular solution. Examples of responding and nonresponding neurons are depicted. (**B**) Time course of mean current densities during control conditions, and in the presence of GDP-β-S. NPS (50 nM) was bath applied for 2.5 min. Of note, the mean current density in the presence of GDP-β-S was calculated from responsive and nonresponsive neurons, since 50 nM NPS elicited a detectable current in only one of six recorded neurons (**E**). For comparison, nonresponsive neurons of the control were included in the analysis here. (**C**) Statistical analysis reveals a lack of effect in GDP-β-S treated neurons upon application of 50 nM NPS. (**D**) Time course of mean current densities during control recordings, and in presence of 2 mM GDP-β-S. NPS (150 nM) was applied for 2.5 min. (**E**) Fractions of responsive and nonresponsive neurons during control conditions (50 nM or 150 nM NPS), and in the presence of GDP-β-S (50 nM or 150 nM NPS). In the presence of GDP-β-S, 50 nM NPS fails to evoke a detectable current compared to control conditions. (**F**) To test for a possible role of the Gβ/γ-subunits, the subunit-specific inhibitor gallein (50 µM) was used. Example trace shows an NPS-evoked current in the presence of gallein. (**G**) Time course of NPS-dependent currents during control conditions, and in presence of 50 µM gallein. (**H**) Quantification of mean maximal current densities evoked by 150 nM NPS in the presence of GDP-β-S or gallein compared to the control. * *p* < 0.05; ** *p* < 0.01.

Activation of the NPSR1 by its ligand NPS triggers intracellular signaling pathways via G_αq_ and/or G_αs_ proteins, as shown in HEK and CHO cells expressing the receptor [1,17,19]. In contrast, the contribution of G protein activity to the induction of the inward current observed here is still unknown. To test the significance of G protein signaling, voltage-clamp recordings in the presence of 2 mM GDP-β-S were performed (Figure 3A). GDP-β-S blocks the activation of G proteins by inhibiting binding of GTP to G proteins [26]. Adding 2 mM GDP-β-S to the intracellular solution prevented responses to 50 nM NPS in aBA PNs (Figure 3B). Only one of six recorded neurons did show a detectable inward current (Figure 3E). To quantify these findings, only here we averaged recorded currents of responsive and non-responsive neurons in the presence and absence of GDP-β-S. Statistical comparison revealed a significant reduction of mean current densities (RM ANOVA: interaction treatment x time: F(2,40) = 3.93; *p* = 0.028; Bonferroni post hoc test: NPS_max_ control vs. NPS_max_ GDP-β-S: *p* = 0.034; baseline control vs. NPS_max_ control: *p*= 8.5 × 10^−7^; baseline control vs. wash control: *p* = 0.006; Figure 3B,C) and a significantly reduced fraction of responsive neurons (Fisher’s exact test: *p* = 0.005; Figure 3E). Application of 150 nM NPS induced a current in 86 % of the recorded neurons in the presence of GDP-β-S (Figure 3E), while the mean current density was significantly reduced to −0.08 ± 0.04 pA/pF in these neurons (*n* = 6; Figure 3H). Next, we used gallein (50 µM) to inhibit G_βγ_ signaling in aBA PNs during application of 150 nM NPS (Figure 3F). Although a trend was observed, the mean current densities were not significantly reduced in the presence of gallein (gallein: −0.48 ± 0.07 pA/pF; *n* = 10; control: −0.667 ± 0.07; *n* = 17; one-way ANOVA: F(2,30) = 9.71; *p*= 5.5×10^−4^; Bonferroni post hoc test: control vs. GDP-β-S: *p* = 4.1 × 10^−4^; gallein vs. GDP-β-S: *p* = 0.047; Figure 3G,H). These data strongly suggest that mostly G_α_ signaling following NPSR1 activation in aBA PNs mediates the NPS-induced current.

### 2.4. The NPSR1-Mediated Current Depends on Rise of Intracellular Calcium Concentrations

**Figure 4:**
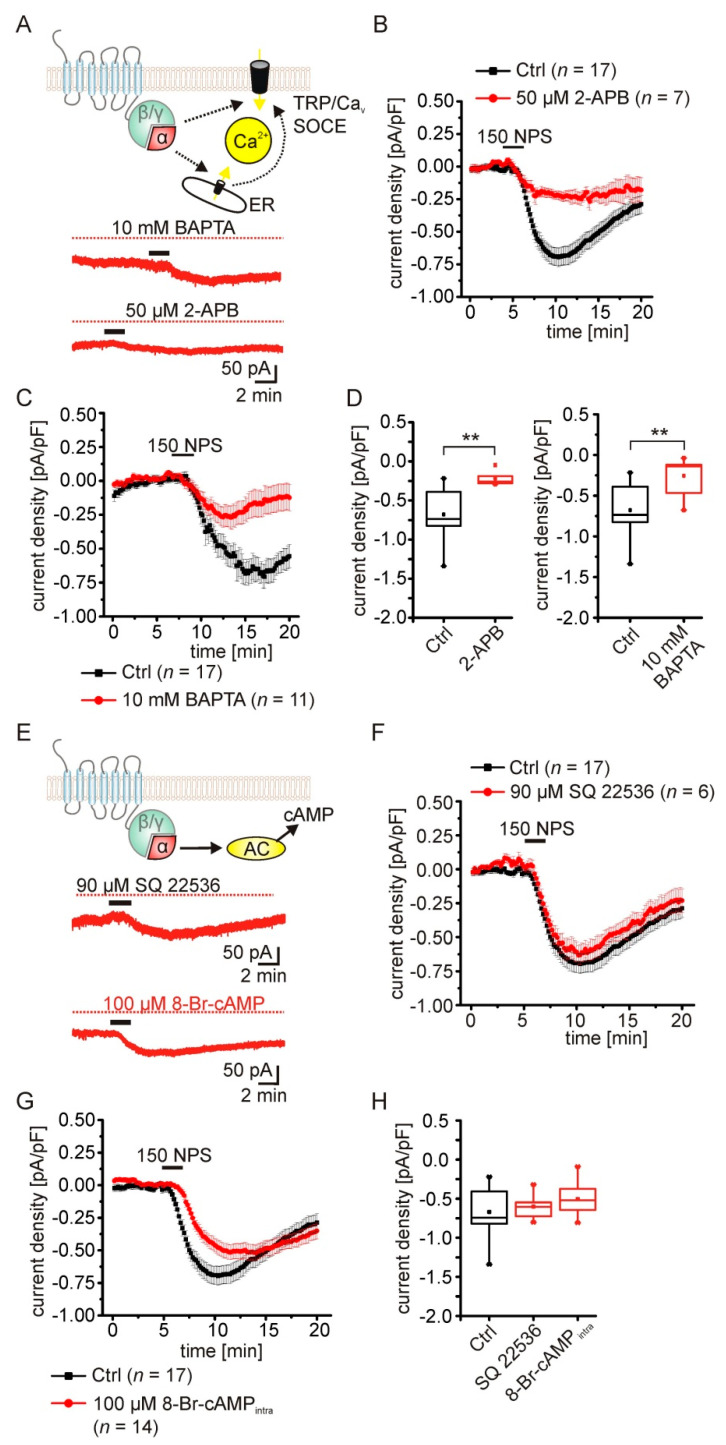
The NPSR1-mediated current depends on a rise of intracellular calcium concentration. (**A**) Activation of the NPSR1 might trigger intracellular signaling cascades leading to a rise of the cytosolic calcium concentration. Putative calcium sources might be the endoplasmic reticulum (ER) or the extracellular space, with TRP channels or store-operated calcium entry (SOCE) as possible routes. (**B**) The transient NPS-induced current is reduced in the presence of 50 µM 2-APB in the ACSF, and during recordings with 10 mM BAPTA in the intracellular solution (**C**). (**D**) The maximal current density in the presence of 2-APB was significantly smaller compared to control conditions. Buffering cytosolic calcium with 10 mM BAPTA significantly reduced the NPSR1-mediated current. (**E**) Following NPSR1 activation, Gαs signaling might trigger cAMP synthesis via adenylyl cyclases (AC). cAMP-dependent kinases might contribute to the observed NPS-dependent current via modulation of potassium conductances. Depicted are example current traces recorded in the presence of AC inhibitor SQ 22536 (90 µM; upper trace) or recorded with 100 µM 8-Br-cAMP in the internal solution (lower trace). Neither SQ 22536 (**F**) nor 8-Br-cAMP (**G**) significantly altered the NPS-evoked inward current. (**H**) Quantification of the maximal current density recorded during control conditions, and in the presence of SQ 22536 or 8-Br-cAMP. No statistically significant changes were observed. ** *p* < 0.01.

According to previous publications, NPSR1 activation increases intracellular calcium and cAMP concentrations via G_αq_ and G_αs_ signaling [1,19]. In order to test the contribution of these signaling cascades to the observed current, we performed voltage-clamp experiments in the presence of 2-APB (Figure 4A). 2-APB has been reported to inhibit inositol−3-phosphate receptors (IP_3_R) in the endoplasmic reticulum (ER), store-operated calcium-entry (SOCE), and a subset of transient receptor potential channels (TRP) in a concentration-dependent manner, but can activate TRPV1, TRPV2, and TRPV3 [27,28,29]. We have shown previously that 2-APB (25 µM) abolishes NPS-induced calcium transients in cultured hippocampal neurons expressing human NPSR1 [19]. Here, 50 µM 2-APB significantly reduced the current induced by 150 nM NPS in aBA PNs of the mouse amygdala (2-APB: −0.219 ± 0.032 pA/pF; *n* = 7; control: −0.667 ± 0.07 pA/pF; *n* = 17; U-Test: U = 4; Z = −3.493; *p* = 0.001; Figure 4B,D). Next, we included 10 mM BAPTA in the recording pipette to buffer cytosolic calcium (Figure 4A,C). BAPTA significantly reduced the NPSR1-mediated current in PNs (BAPTA: −0.256 ± 0.07 pA/pF; *n* = 11; control: −0.679 ± 0.07 pA/pF; *n* = 17; U-test: u = 18; z = −3.53; *p*= 4.2 × 10^−4^; Figure 4C,D). These findings indicate the involvement of intracellular G_αq_-dependent Ca^2+^ signaling following NPSR1 activation.

Next, we tested the possible role of G_αs_ signaling by inhibiting adenylyl cyclase activity with SQ 22536 ([30]; 90 µM; Figure 4E,F). SQ 22536 had no effect on the NPSR1-mediated current elicited by 150 nM NPS (SQ22536: −0.601 ± 0.068 pA/pF; *n* = 6; control: −0.667 ± 0.07 pA/pF; *n* = 17; Figure 4F,H). In addition, adding 100 µM 8-Br-cAMP to the intracellular recording solution to occlude potential NPSR1-dependent cAMP-signaling had no effect on the NPS-evoked current (8-Br-cAMP: −0.504 ± 0.056 pA/pF; *n* = 14; one-Way ANOVA control vs. SQ 22536 vs. 8-Br-cAMP: F(2,34) = 1.71; *p* = 0.195; Figure 4H). These data indicate that the NPSR1-mediated inward current depends on intracellular Ca^2+^ signaling pathways, with no involvement of cAMP-dependent mechanisms. 

### 2.5. Pharmacological Characterization of the NPSR1-Modulated K^+^ Conductance

**Figure 5:**
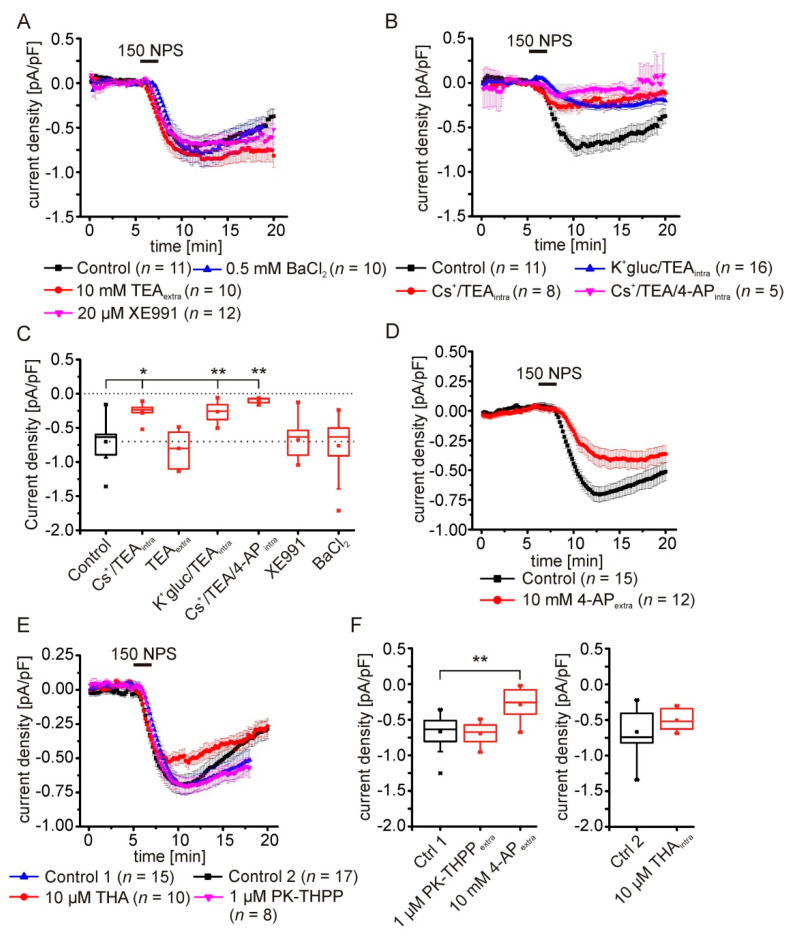
Pharmacological characterization of NPSR1-modulated potassium conductances. (**A**) Neither the time course nor the maximal amplitude of the NPSR1-mediated current elicited by 150 nM NPS are altered by 0.5 mM BaCl_2_, 10 mM extracellular TEA, or 20 µM XE991. (**B**) The NPSR1-mediated current is sensitive to intracellular recording solutions containing: K^+^-gluconate and 10 mM TEA (blue), Cs^+^-methylsulfonate and 10 mM TEA (red), and Cs^+^-methylsulfonate, 10 mM TEA, and 10 mM 4-AP (magenta). (**C**) Quantification of the mean current densities depicted in (**A**) and **(B**). (**D**) In the presence of 10 mM 4-AP, the mean current density of the NPSR1-mediated current is significantly attenuated. (**E**) Intracellular THA (10 µM) or extracellular PK-THPP (1 µM) do not affect the NPSR1-mediated current. (**F**) Quantification of the current densities depicted in D) and E). 10 mM 4-AP, but not PK-THPP, significantly reduces the NPSR1-mediated current (one-way ANOVA: F(2,32) = 12.57; *p* = 9.3 × 10^−5^; post hoc test: 4-AP vs. ctrl 1: *p* = 2.5 × 10^−4^). Intracellular THA (10 µM) does not significantly affect the NPSR1-mediated current (*t*-test: *t* = −1.67; df = 25; *p* = 0.107). * *p* < 0.05; ** *p* < 0.01.

In the next series of experiments, we further characterized the K^+^ conductance evoked by NPSR1 stimulation. We applied tetraethyl-ammonium (10 mM) via the ACSF to inhibit a broad spectrum of delayed-rectifier K^+^ channels, XE991 (20 µM) to inhibit M-currents generated by members of the KCNQ channel family, and BaCl2 (0.5 mM) to inhibit classes of inward-rectifier and G-protein-dependent inward-rectifier (G_irk_) K^+^ channels (Figure 5A,C). None of these extracellularly applied compounds inhibited the NPSR1-mediated current upon application of 150 nM NPS. However, using a cesium-based intracellular solution containing 10 mM TEA significantly reduced the NPS-elicited current (Cs_intra_: −0.257 ± 0.04 pA/pF; *n* = 8; control: −0.703 ± 0.09 pA/pF; *n* = 11; Figure 5B,C). To differentiate between inhibition of K^+^ channels by cesium or intracellular TEA, we used a K^+^-gluconate-based recording solution containing 10 mM TEA. Intracellular TEA alone reduced the NPSR1-mediated current significantly (K-gluc-TEA_intra_: −0.264 ± 0.04 pA/pF; *n* = 16; control: −0.703 ± 0.09 pA/pF; *n* = 11; Figure 5B,C).

In order to achieve additional inhibition of TEA-resistant K^+^ channels, i.e., channels contributing to transient outward currents (KCND1-3), we next used a cesium-based intracellular solution containing TEA and 4-aminopyridine. Using this intracellular solution, the NPSR1-mediated current was barely detectable (Cs/TEA/4-AP: −0.101 ± 0.02 pA/pF; *n* = 5; control: −0.703 ± 0.09 pA/pF; *n* = 11; one-way ANOVA: F(6,65) = 10.56; *p* = 3.7 × 10^−8^; post hoc test: control vs. Cs+/TEA_intra_ *p* = 0.013; control vs. K^+^-gluc/TEA_intra_ *p* = 0.002; control vs. Cs+/TEA/4-AP_intra_ *p* = 0.002; Figure 5B,C). Next, recordings with a K^+^-gluconate-based solution were performed in ACSF containing 10 mM 4-AP. 4-AP alone reduced the NPSR1-mediated current significantly to −0.286 ± 0.067 pA/pF (*n* = 12) compared to controls (−0.668 ± 0.061 pA/pF; *n* = 15; Figure 5D,F).

In a final series, we tested inhibitors of TASK channels that belong to the two-pore domain K^+^ channel (K2P) family. Neither intracellular 10 µM tetrahexylammonium chloride (THA), nor extracellular PK-THPP (1 µM; TASK1/3-specific) did modulate the NPSR1-mediated current significantly (PK-THPP: −0.695 ± 0.056; *n* = 8; control 1: −0.668 ± 0.061; *n* = 15; THA: 0.504 ± 0.045 pA/pF; *n* = 10; control 2: −0.669 ± 0.071; *n* = 17; Figure 5E,F). Our detailed characterization revealed that the NPSR1-mediated current is sensitive to intracellular TEA and 4-AP, indicative for voltage-gated potassium channels.

### 2.6. NPSR1 Activation Enhances Action Potential Generation in aBA PNs

**Figure 6:**
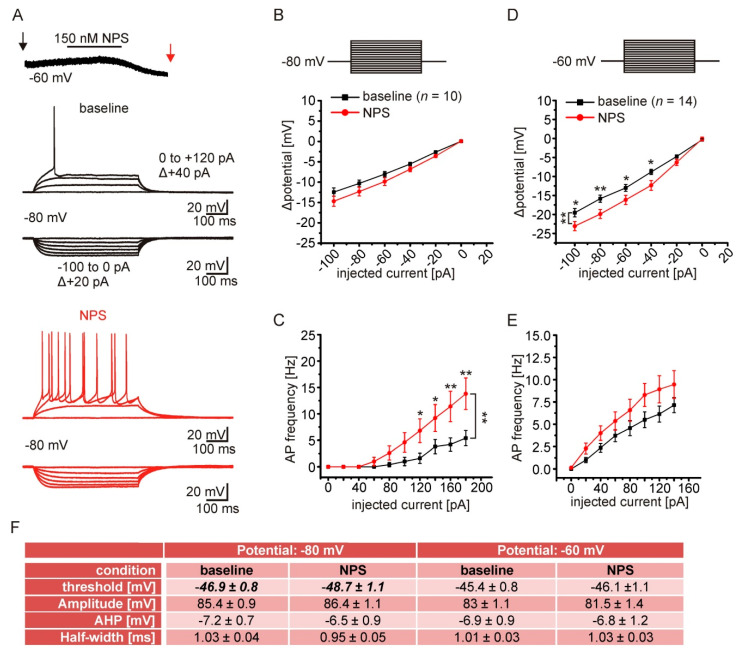
NPSR1-activation enhances action potential generation in aBA PNs. (**A**) Example of the experimental procedure. Current-clamp recordings were done during baseline conditions (black arrow) and during maximal NPS effect (red arrow). The onset of the NPS effect was monitored in voltage-clamp at −60 mV. In current-clamp, hyper- and depolarizing current injections (first: −100 pA; Δ + 20 pA; 500 ms duration) were applied from a membrane potential of either −60 mV or −80 mV. Example traces depict recordings from −80 mV during baseline (black) or in the presence of NPS (red). (**B**) Analysis of passive membrane properties during current injections (−100 to + 50 pA) from a membrane potential of −80 mV. No significant changes of Δpotential in response to current injections were observed in the presence of NPS. (**C**) Frequency of evoked action potentials is enhanced in the presence of NPS compared to baseline. (**D**) At −60 mV hyperpolarizing current injections resulted in enhanced Δpotential in the presence of NPS. (**E**) At −60 mV depolarizing current injections did not change the discharge pattern significantly. (**F**) Analysis of action potential characteristics during baseline conditions and in the presence of NPS during both recording conditions. * *p* < 0.05; ** *p* < 0.01.

NPSR1-dependent reduction of K^+^ conductances likely affects the excitability of aBA PNs. To test this possibility, we performed current-clamp recordings during baseline conditions and during near-maximal NPS effects (Figure 6A). Passive and active membrane properties were analyzed by injecting hyper- and depolarizing rectangular current pulses (500 ms duration), with step-wise (+20 pA) increases in amplitude from a membrane potential of either −80 or −60 mV. The mean resting membrane potential of aBA PNs was at −75 ± 1 mV, and the mean membrane capacitance was 64 ± 5 pF (*n* = 14). Next, hyperpolarizing currents were injected from a membrane potential of −80 mV. The input resistance during control and in the presence of NPS was calculated from the slope of the I–V plots (R_in_ = ΔU/ΔI). Although the change of the membrane potential in response to hyperpolarizing current injections from −80 mV was not significantly different in the presence and absence of NPS (Figure 6B), the calculated input resistance was significantly enhanced in the presence of NPS. R_in_ was 132 ± 12 MΩ during control conditions, and increased to 153 ± 14 MΩ in the presence of NPS (paired *t*-test: *t* = −4.56; df = 9; *p* = 0.001). In addition, action potential generation in response to depolarizing currents was enhanced in the presence of NPS (RM-ANOVA: interaction treatment x current F(6108) = 4.87; *p* = 1.9 × 10^−4^; LSD post hoc test: baseline vs. NPS 120 pA: *p* = 0.042; 140 pA *p* = 0.035; 160 pA *p* = 0.006; 180 pA *p* = 0.002; *n* = 10; Figure 6C). Concomitantly, we observed a negative shift of the action potential threshold during NPSR1 application, while other active membrane properties were unchanged (threshold_control_ vs. threshold_NPS_: paired *t*-test: *t* = 2.63; df = 9; *p* = 0.027; *n* = 10; Figure 6F).

Furthermore, we observed significant effects of NPS application for hyperpolarizing current injections at −60 mV. Larger changes of the membrane potential in the presence of NPS were found, thereby indicating an increase in the input resistance (RM-ANOVA interaction treatment x current F(5,130) = 4.29; *p* = 0.001; LSD post hoc test: baseline vs. NPS: −100 pA *p* = 0.01; −80 pA *p* = 0.003; −60 pA *p* = 0.021; −40 pA *p* = 0.01; *n* = 14; Figure 6D). The mean input resistance calculated from the I–V plots was 192 ± 12 MΩ during baseline conditions, and increased to 268 ± 24 MΩ in the presence of NPS (paired *t*-test: *t* = −3.061; df = 13; *p* = 0.009; *n* = 14). In contrast, NPS failed to significantly increase neuronal discharges in response to depolarizing current injections at -60 mV (RM-ANOVA: treatment vs. current F(7175) = 1.25; *p* = 0.28; *n* = 14; Figure 6E). These data show that NPSR1 activation enhances neuronal discharges, accompanied by a reduction of the action potential threshold and a moderate increase of input resistance.

## 3. Discussion

The vast majority of available studies describe the function of the NPS system on a network level [8,22], analyze behavioral consequences following pharmacological or genetic interventions [5,9,31], or point out implications of NPSR1 single-nucleotide polymorphisms for psychiatric disorders in humans [32,33]. Other studies analyzed structure–function relationships of NPSR1 and NPS, using HEK or CHO expression systems and rodent models [24,34,35]. In contrast, little is known about signaling cascades in neurons endogenously expressing the NPSR1.

Here, we used electrophysiological approaches, combined with pharmacology, to investigate intracellular signaling and possible second messengers and effectors in principal neurons of the anterior basolateral complex of mice. Based on our data, we can (a) confirm the expression profile of NPSR1 mRNA in the aBA, (b) describe NPS-specific activation of the NPSR1 in aBA PNs of male and female mice, (c) provide evidence that the NPSR1-mediated inward current is dependent on Gαq signaling, (d) identify a reduction of membrane K^+^ conductance in these neurons as consequence of NPSR1 stimulation, and (e) show NPS-induced increase in neuronal excitability.

As described previously [3,8,24], high NPSR1 mRNA expression can be detected in the aBA of mice, whereas it is almost absent in the posterior BA or the central nuclei of the amygdala. NPSR1 activation by the application of NPS induced a transient inward current in about 80 to 90 % of the recorded aBA PNs, which was abolished by the NPSR1-specific antagonist SHA-68 [25]. The NPSR1-mediated current was not different between males and females, in line with our previous findings documenting a lack of sex-based differences on the cellular level [24]. This inward current was accompanied by an increase in apparent input resistance, which has also been described in projection neurons of the endopiriform nucleus [22]. These findings indicate a reduction of membrane conductance, which is active at a membrane potential of −60 mV. Analysis of current–voltage relationships of the NPS-induced current and recordings in elevated extracellular K^+^ concentrations revealed a reversal potential near the respective K^+^ equilibrium potential, identifying K^+^ ions as carriers of the current. GPCRs can modulate K^+^ channel activity through changes in channel conductance and/or open probability. For example, activation of muscarinic receptors induces G-protein-mediated negative modulation of K2P channels involving different intracellular signaling molecules [36,37] in, e.g., thalamic relay neurons.

The NPSR1-mediated current requires intracellular G protein activity, as inhibition by GDP-β-S abolished the inward current. Thus, direct protein–protein interaction of the NPSR1 and a putative effector seems not to be involved. Furthermore, inhibition of the β/γ-subunits by gallein had no effect on the NPSR1-mediated current, leading to the conclusion that Gα-signaling is mandatory. Depending on the α-subunit involved (αs and/or αq), different downstream signaling molecules, such as adenylyl cyclases (AC; by G_αs_) or phospholipases (PL; G_αq_), would be activated in prototypic pathways. Here, NPSR1-mediated currents were insensitive to AC inhibition or intracellular cAMP application, ruling out a major contribution of Gαs signaling, which has been described to occur after NPSR1 activation in HEK or CHO cells [17,38]. In contrast, the NPSR1-mediated current in aBA PNs was strongly reduced by manipulation of intracellular Ca^2+^ by either BAPTA or 2-APB. Intracellular Ca^2+^-transients have been described in detail previously in cultured hippocampal neurons expressing human NPSR1 [19]. In these assays, 2-APB abolished the rise of intracellular Ca^2+^, most likely due to inhibition of IP3Rs and SOCE. In addition, in dorsal raphe (DR) and laterodorsal tegmentum (LT), NPS application induced an increase of intracellular Ca^2+^ involving IP3Rs and RyR [21]. Moreover, the NPSR1-mediated current was also dependent on Ca^2+^ in DR and LT neurons. Thus, mobilization of intracellular Ca^2+^ as a second messenger seems to be shared by aBA PNs and DR/LT neurons following NPSR1 activation. It is interesting to note that Ca^2+^ release induced by PLC activation feeds back on PLC, a strong positive feedback mechanism that is sensitive to BAPTA application [39], thereby emphasizing the significance of the G_αq_/PLC pathway for the present findings.

While the involvement of K^+^ channels seems to be clear, our pharmacological assays provide some hints with respect to K^+^ channel isoforms modulated by NPSR1, without identifying the exact types of channels involved. Results obtained with barium at the concentration used in the present study exclude the involvement of G-protein-coupled inward rectifier (G_irk_) channels as potential targets. The concentration of XE991 used inhibits KV7.1/2/4, but not KV7.5, which has been shown to mediate M-type currents in various neurons of various brain regions, including BLA [40]. Overall, the NPS-evoked K^+^ current was sensitive to intracellular TEA and 4-AP, typifying delayed rectifier and A-type K^+^ channels. 4-AP-sensitive K^+^ channels of the KCND family mediate a transient, hyperpolarization-dependent A-type current, which is thought to mediate a delayed onset of spike firing if evoked from membrane potentials negative from resting membrane potentials [41]. The lack of noticeable delayed onset of firing in our current-clamp recordings from −80 mV suggests that A-type currents are not prominently present in aBA PNs, and are unlikely to mediate the NPSR1-mediated current. The lack of effect of THA or PK-THPP excludes TASK channels as targets of NPSR1 signaling [42]. Taken together, these data leave members of the delayed rectifier classes and Kv7.5 as most plausible candidates. M-current mediating K_V_7.5 channels are inhibited directly by intracellular Ca^2+^ [43] or, e.g., by A-kinase-anchoring protein AKAP150 [44] following GPCR-activation. Thus, NPSR1-dependent intracellular signaling cascades, including Ca^2+^, could negatively modulate K_V_7.5 channels. Of note, we cannot completely rule out additional modulations of voltage-gated Ca^2+^ channels in aBA PNs by NPSR1 activation [45,46]. Future experiments using elaborated pharmacology and/or single-cell mRNA sequencing are needed to identify the exact target channels. Delayed rectifier channels of the Kv2.1 subtype, characterized by PLC-mediated regulation, 4-AP-sensitivity, and incomplete inactivation, are promising candidates [47,48].

In summary, we show that NPSR1 activation by NPS increases neuronal excitability in aBA PNs by the inhibition of voltage-gated K^+^ channels. We provide data indicating the involvement of G_αq_- and intracellular calcium signaling in these neurons. As a functional consequence, NPS release in aBA will increase local network activity and can thereby modulate information processing in emotion-relevant circuits between aBA and the posterior BA (pBA), as shown before [24,49,50].

Thus, the NPS/NPSR1 system is apt to shape emotional states in response to aversive stimuli and to modulate threat responses during expression of learned fear and fear extinction [7,8,24]. In turn, alterations of NPSR1 signaling efficacy by nonsynonymous mutations in the NPSR1 [24,32,33,51] or the NPS gene [51] might consequently alter behavior and the risk for the development of psychiatric disorders. It is evident that detailed knowledge about neuropeptide systems and underlying signaling cascades is important to understand their impact on the development of psychiatric disorders. An increasing number of different neuropeptide systems (e.g., galanin, cholecystokinin, and NPY) have been shown to modulate fear [52,53,54,55] and anxiety [56,57,58] in rodents and humans [59]. In this respect, it is interesting to note that orexin, an excitatory neuropeptide with well-described roles in regulation of arousal and energy homeostasis, was recently found to modulate fear responses [60]. The pathway underlying in this modulation involved the PLC-dependent depolarization of neurons in the central nucleus of the amygdala, thereby pointing to some similarities in neuropeptide signaling pathways and the in vivo relevance of their modulatory influence. Therefore, detailed analysis of GPCR signaling, effects of human-relevant polymorphisms in neuropeptide systems, and the interplay between different neuropeptide systems is needed to understand and possibly treat psychiatric diseases.

## 4. Materials and Methods

All animal experiments were carried out in accordance with European regulations on animal experimentation (European Committee Council Directive 2010/63/EU; National Research Council of the National Academies), and approved by the local authorities (LANUV).

### 4.1. Animals

C57BL/6N were kept in a temperature-controlled (21 °C) and humidity-controlled (50–60 % relative humidity) animal facility in individually ventilated cages, with access to food and water ad libitum and a 12 h light/dark cycle, with lights on at 6:00 am. Food, water, and animal conditions were controlled on a daily basis. For experiments, male and female mice 6 to 8 weeks of age were used.

### 4.2. Electrophysiology

Experimental procedures were carried out as described previously [24]. Mice were decapitated, and brains were quickly removed. Coronal or horizontal brain slices (300 µm thickness), containing the amygdala, were cut on a vibratome (VT1200S; Leica, Germany). Slices were placed in a submersion chamber at 30 °C and were perfused with artificial CSF (ACSF) containing the following (in mM): 120 NaCl, 2.5 KCl, 1.25 NaH_2_PO_4_, 2 MgSO_4_, 2 CaCl_2_, 22 NaHCO_3_, and 20 glucose. The pH was set to 7.35 by gassing with carbogen. Perfusion speed was set to 3.5–4 mL/min.

Patch pipettes (2.5–4 MΩ pipette resistance) were made of borosilicate glass (GC150T-10; Harvard Apparatus, Holliston, MA, USA). The intracellular solution contained the following (in mM): 10 NaCl, 105 potassium gluconate, 20 potassium citrate, 10 HEPES, 3 BAPTA, 0.5 CaCl_2_, 1 MgCl_2_, 3 MgATP, 0.5 NaGTP, and 15 phosphocreatine, pH adjusted to 7.25. The liquid junction potential of 10 mV was compensated online. In some experiments, a Cs^+^-based intracellular solution was used, containing (mM): 5 4-aminopyridine, 120 CsMeSO_4_, 1 EGTA, 10 HEPES, 20 tetraethylammonium chloride, 2 MgCl_2_, 0.5 CaCl_2_, 2 Na-ATP, and 0.5 Na GTP. To analyze NPS-induced currents, aBA principal neurons (PN) were recorded in the voltage-clamp mode at a membrane potential of −60 mV. Neurons were recorded for at least 15 min. Every 10 s, a voltage-step of −5 mV was used to monitor the apparent input resistance. To reduce network activity, the ACSF contained: tetrodotoxin citrate (TTX; 0.5 µM; Abcam, Cambridge, UK), 6,7-dinitroquinoxaline-2,3-dione disodium salt (DNQX; 10 µM; Abcam), DL-2-amino-5-phosphonopentanoic acid sodium salt (AP-5; 25 µM; Abcam), 2-(3-carboxypropyl)-3-amino-6-(4 methoxyphenyl)pyridazinium bromide (gabazine; 10 µM; Abcam), and (2*S*)-3-[((1*S*)-1-(3,4-dichlorophenyl)ethyl)-amino-2-hydroxypropyl]-(phenylmethyl)phosphinic acid hydrochloride (CGP55845; 2.5 µM; Tocris). NPS (50 or 150 nM; Tocris, Bristol, UK) was bath-applied for 2.5 min. The recorded current was normalized to the capacitance of the PN and is presented as current density (pA/pF) to correct for differences in PN size. Recorded aBA PNs were sorted into responsive and nonresponsive by statistical comparison (paired *t*-test) of current densities during baseline, with current densities in a 2.5 min time window after NPS application. If not stated otherwise, only responsive PNs were analyzed. In total, four sets of control recordings were obtained. Pharmacological interventions are compared to the respective set of control recordings.

Voltage-clamp step protocols were performed from a holding potential of −60 mV, and steps from −120 mV to −50 mV (Δ + 10 mV) were applied for two seconds each. Resultant current traces during baseline and in the presence of NPS were subtracted (current_NPS_–current_baseline_) to calculate the NPS-induced (NPSR1-mediated) current. The reversal potential of the NPS-induced current was calculated for each individual PN. Hyperpolarizing voltage-clamp ramps were done from a step to 0 mV (1 s) to −120 mV (ramp velocity: 0.48 mV/ms). The NPS-induced current was calculated from ramp_NPS_ and ramp_baseline_, analyzed as described above. Substances for pharmacological interventions: *n*-[((4-fluorophenyl)methyl)tetrahydro-3-oxo-1,1-diphenyl-3H-oxazolo(3,4-a)]pyrazine-7 (1*H*)-carboxamide (SHA-68; 10 µM; Tocris, Bristol, UK), GDP-β-S trilithium salt (2 mM; Sigma Aldrich, St. Louis, MO, USA), 3′,4′,5′,6′-tetrahydroxyspiro[isobenzofuran-1(3H),9′-(9H)xanthen]-3-one (Gallein; 50 µM, Tocris), 1,2-bis(2-aminophenoxy)ethane-N,N,N′,N′-tetraacetic acid (BAPTA, 10 mM, Sigma Aldrich, St. Louis, MO, USA); 2-aminoethoxydiphenylborate (2-APB; 50 µM; Sigma Aldrich, St. Louis, MO, USA), 8-bromoadenosine 3′,5′-cyclic monophosphate sodium salt (8-Br-cAMP; 100 µM; Sigma Aldrich, St. Louis, MO, USA), 9-(tetrahydro-2-furanyl)-9H-purin-6-amine (SQ 22536; 90 µM; Tocris, Bristol, UK), 10,10-bis(4-pyridinylmethyl)-9(10H)-anthracenone dihydrochloride (XE991; 20 µM; Tocris, Bristol, UK), tetraethylammonium chloride (TEA; 10 mM; Sigma Aldrich, St. Louis, MO, USA), 4-aminopyridine (4-AP; 10 mM; Sigma Aldrich, St. Louis, MO, USA), tetrahexylammonium chloride (THA; 10 µM; St. Louis, MO, USA), 1-[1-[6-((1,1′-biphenyl)-4-ylcarbonyl)-5,6,7,8-tetrahydropyrido(4,3-d)pyrimidin-4-yl]-4-piperidinyl]-1-butanone (PK-THPP; 1 µM; Tocris, Bristol, UK). To inhibit adenylyl cyclases, slices were preincubated in ACSF containing SQ 22536 for >1 h, and SQ 22536 was included in the pipette during subsequent recordings. The concentration of the anorganic solvent dimethylsulfoxide (DMSO) was kept below 0.02 % when present. DMSO alone at the concentration used did not interfere with the observed NPSR1-mediated current.

Hyper- and depolarizing current injections (500 ms duration; Δ +20 pA) were performed in the current-clamp mode at membrane potentials of −60 and −80 mV. For current–voltage relationships, shifts of the membrane potential (Δpotential) in response to hyperpolarizing current were analyzed. NPS was applied at a concentration of 150 nM, and the onset of the NPS-induced inward current was monitored in voltage-clamp mode. All electrophysiological recordings were performed using a HEKA 10 double patch clamp amplifier and PatchMaster software.

### 4.3. RNAScope

RNAScope (ACD Biotechne, Newark, CA, USA) was used to detect *Npsr1* mRNA in aBA neurons via fluorescence in situ hybridization. All steps were performed according to the manufacturer’s instructions. Coronal sections containing the amygdala were prepared from fresh frozen brain tissue. Detection of *Npsr1* mRNA was performed in 16 µm thick fresh frozen coronal slices of, in total, two animals, using RNAScope Multiplex-Fluorescent assay kit with predesigned probes for mouse *Npsr1*. Stained specimens were covered with Vectashield mounting medium (Vector Laboratories, Burlingame, CA, USA) and analyzed using a confocal microscope (Nikon, Minato, Japan) with appropriate objectives and filter sets (DAPI: 450/35; Alexa488: 515/30; Atto550: 605/75). Image stacks (7 µm *z*-axis; 1 µm steps) were acquired (1024 × 1024 resolution) using an Achromat 16.0 x/0.80/3.00 NCG Water Dip objective. Image stacks were processed with ImageJ 1.52p (Wayne Rasband, National Institute of Health, Bethesda, MD, USA). Using maximum z-projection, stacks were merged into a 2D picture.

### 4.4. Analysis and Statistics

All datasets were retrieved from neurons of at least three mice. The number of experiments “*n*” represents the number of analyzed neurons. Electrophysiological recordings were analyzed offline using Clampfit 10.5 (Molecular Devices, San Jose, CA, USA). Statistical analysis was done with Origin 9.1 (Origin Lab Corporation, Northampton, MA, USA) and Statistica 64 (Tibco Software Inc., Palo Alto, CA, USA). All final datasets were tested once for statistical outliers using Grubb’s test, and identified values were removed. Statistical comparison was done using either Student’s *t*-test or Mann–Whitney U-test, depending on normal or non-normal data distribution. One-way ANOVA or repeated measurements ANOVA (RM-ANOVA) were used and Bonferroni or Fisher’s LSD post hoc tests were applied as indicated. CorelDraw X4 (Corel Coperation, Ottawa, ON, Canada) was used for data presentation. Statistically significant differences are presented as: * *p* < 0.05; ** *p* < 0.01.

## Data Availability

The data presented in this study are available on request from the corresponding author.
